# Confounders of severe asthma: diagnoses to consider when asthma symptoms persist despite optimal therapy

**DOI:** 10.1186/s40413-018-0207-2

**Published:** 2018-11-14

**Authors:** Alina Gherasim, Ahn Dao, Jonathan A Bernstein

**Affiliations:** 1 Hôpitaux Universitaires de Strasbourg, Strasbourg, France; 2 University of Cincinnati College of Medicine, Cincinnati, OH USA; 3 Department of Internal Medicine, Division of Immunology Rheumatology and Allergy, University of Cincinnati, 231 Albert Sabin Way ML#563, Cincinnati, OH 45267-0563 USA

## Abstract

Asthma can often be challenging to diagnose especially when patients present with atypical symptoms. Therefore, it is important to have a broad differential diagnosis for asthma to ensure that other conditions are not missed. Clinicians must maintain a high index of suspicion for asthma mimickers, especially when patients fail to respond to conventional therapy. The purpose of this review is to briefly review some of the more common causes of asthma mimickers that clinicians should consider when the diagnosis of asthma is unclear.

## Background

Previous studies have suggested an overdiagnosis of asthma in up to one third of individuals [1, 2]. The aim of this paper is to review other diseases that can masquerade asthma and serve as a reminder to Chevalier Jackson’s famous quote, “all that wheezes is not asthma” [3].

**Fig. 1:**
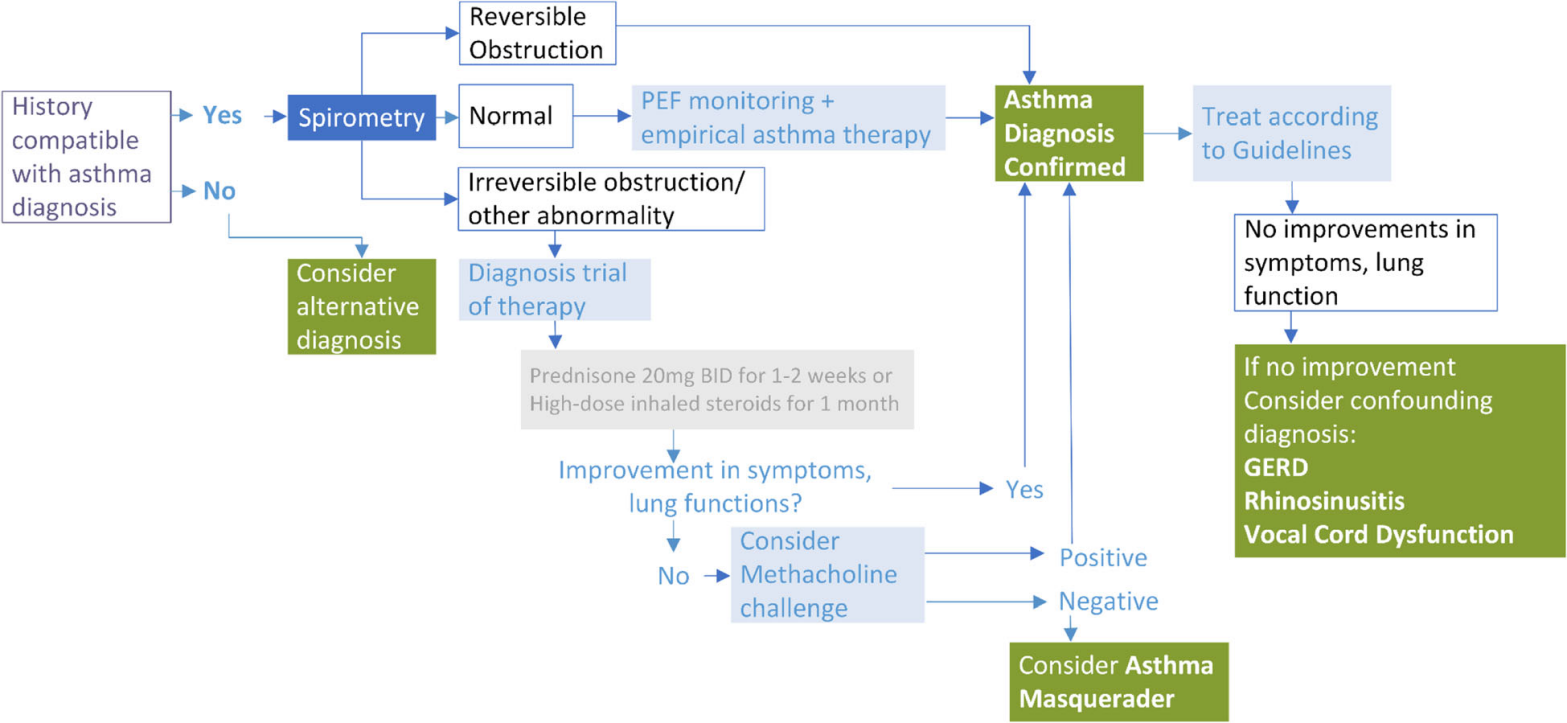
Algorithmic Evaluation of Asthma

The definition of asthma has been an ongoing subject of debate. This is, in part, related to its heterogeneous presentations as a variety of phenotypes. The Global Initiative in Asthma (GINA) task force has defined asthma as, “a heterogeneous disease, usually characterized by chronic airway inflammation defined by the history of respiratory symptoms such as: wheeze, cough, chest tightness and shortness of breath that vary over time and intensity, together with airway limitation” [4]. A definitive diagnosis is obtained by objective testing that includes: 1) spirometry where there is evidence of obstruction (FEV1/FVC < 70%) with at least a 12% or 200 mL increase in FEV1 post-bronchodilator; 2) positive methacholine bronchoprovocation test where there is a 20% fall in the FEV1 (or an equivalent bronchoprovocation test that meets the cut-off criteria for being a positive test)or; 3) more than 20% peak flow variability over time. The negative predictive value is the most useful aspect of the methacholine challenge [4, 5]. In adults, a significant increase in lung function (> 200 ml) after 4 weeks of an oral or inhaled anti-inflammatory treatment should also be considered an asthma diagnostic feature [5]. An algorithm summarizing the approach for asthma diagnosis is illustrated in Fig. 1 [6].

Once a diagnosis of asthma is established, studies have shown that poor inhaler technique can lead to poor asthma outcomes [7–11]. Therefore, for those patients not responding to asthma treatment, prior to considering other underlying causes that could be obfuscating their clinical improvement, it is first important to confirm that the patient is properly using their asthma inhalers.

**Fig. 2:**
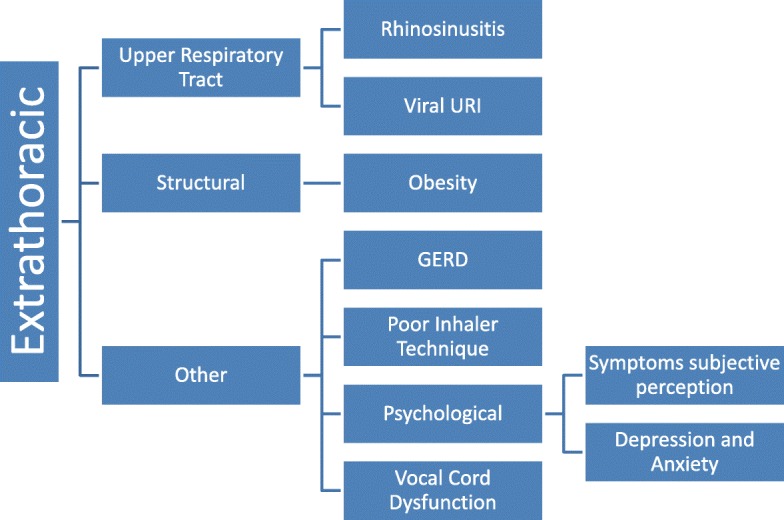
Differential diagnosis of extrathoracic mimickers of asthma

**Fig. 3:**
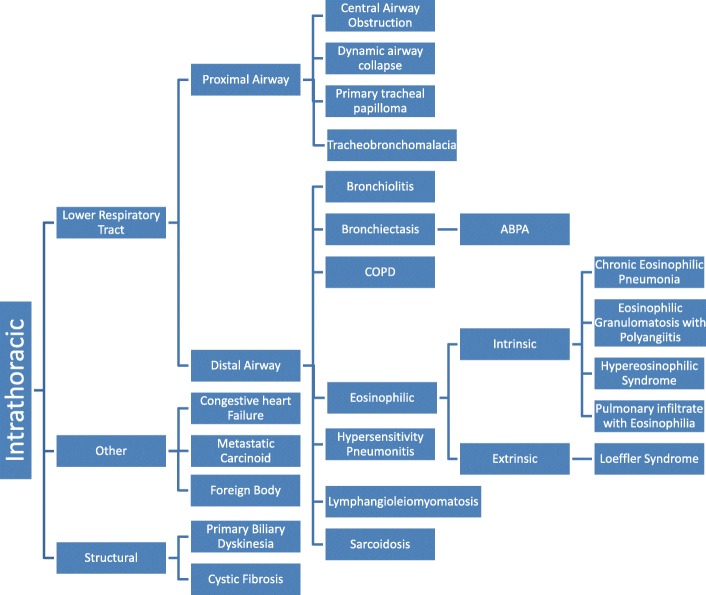
Differential diagnosis of intrathoracic mimickers of asthma

However, patients with asthma or presenting with asthma symptoms not responding to medications may have other causes that could be interfering with their clinical improvement. These conditions can be classified as extrathoracic (Fig. 2) or intrathoracic (Fig. 3). The purpose of this review is to summarize some of the more common causes of asthma mimickers that clinicians should consider when the diagnosis of asthma is unclear or patients with confirmed asthma are not responding to medications as expected. Many conditions may be confounded by or associated with asthma, and the latter can influence clinical manifestations of a patient. The most likely conditions to be mistaken for asthma in clinical practice are discussed in detail in this review. For the less common and uncommon asthma mimickers, including those that also may aggravate or delay the diagnosis, only their typical clinical presentation and the basic diagnostic approach for each condition is summarized.

There is an increasing importance to carefully perform a differential diagnosis for patients presenting with severe asthma symptoms and to reevaluate all potential asthma triggers and inducers each time a step-up-therapy is considered by asking the question: “Is it asthma?”

## Extrathoracic mimickers of asthma

### Upper Respiratory Tract: Viral Upper Airway Infections and Rhinosinusitis

Upper respiratory tract infections (URI) and rhinosinusitis are common causes of coughing. Typically, URIs are caused by rhinoviruses and do not cause chronic symptoms (cough lasting > 8 weeks). The natural course of the common cold usually means that the cough is improved by 2 weeks and steadily resolves by three to 4 weeks [12, 13]. However, an increased viral load can contribute to airway inflammation and lead to an asthma exacerbation [14, 15]. Human rhinoviruses are not only the main pathogens responsible for the common cold but now are recognized to have a major impact on asthma pathogenesis. There is evidence that rhinovirus infections play a role in asthma development as they are a major cause of early childhood wheezing illnesses, asthma exacerbations and, potentially, airway remodeling. The phenomenon following early respiratory syncytial virus (RSV) lower respiratory tract infections of recurrent episodes of wheezing may be a mimicker of early childhood asthma during childhood [16]. This is especially true given that bronchial hyperresponsiveness can be observed for 6 weeks after a viral upper respiratory infection in non-asthmatic patients [17].

Asthma is often associated with comorbidities such rhinitis or sinusitis. Chronic rhinosinusitis with and without nasal polyps, may aggravate some symptoms, particularly cough, which may be attributed to severe asthma [18]. In patients where chronic rhinitis is suspected, it is important to start treatment with an intranasal corticosteroid to see if symptoms improve and if it accelerates recovery, as rhinitis practice parameters recommend this medication as being the most effective therapy for treatment of allergic rhinitis [19]. However, nasal corticosteroids alone are not as effective in treating nonallergic rhinitis conditions such as non-allergic rhinitis with eosinophilia (NARES) or vasomotor rhinitis, so the addition of an intranasal antihistamine may be necessary to improve treatment response in these patients. Typically, improvement in symptoms can be seen in a few days but can take up to 2 weeks to achieve a maximal response [20, 21].

### Vocal Cord Dysfunction

**Fig. 4:**
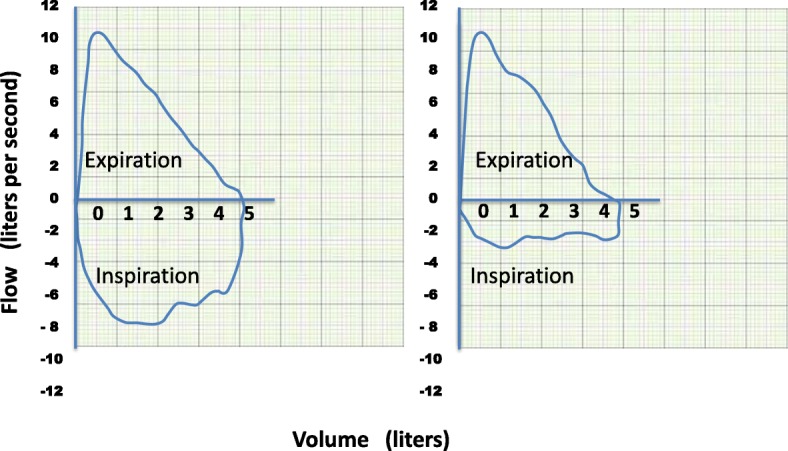
Normal flow volume loop (left) and flow volume loop of a patient with extrathoracic obstruction with vocal cord dysfunction (VCD) (right)

Vocal cord dysfunction (VCD) is a heterogeneous disorder that involves paradoxical inappropriate motion of the true vocal cords, usually seen as episodic unintentional adduction of the vocal cords on inspiration. Typically, this results in dyspnea and stridor on inspiration. However, asthma is associated more often with expiratory wheezes. Spirometry is normal with VCD except during active episodes where there may be flattening of the inspiratory loop consistent with extrathoracic airway obstruction (Fig. 4). Diagnosis can be confirmed with visualization of abnormal vocal cord adduction on laryngoscopy or by videostroboscopy [22]. Acute management includes reassurance and supportive care until the episode resolves spontaneously. Panting can abort episodes as it activates the cricoarytenoid muscle causing abduction of the true vocal cords [23]. Long term treatment focuses on episode prevention by minimizing laryngeal irritation due to gastroesophageal reflux combined with voice exercises by a speech language pathologist, focusing on laryngeal relaxation techniques [24]. Appropriate treatment of associated conditions such as anxiety or depression can also improve the symptoms. Vocal cord dysfunction primarily affects women and adolescent girls and there are two main phenotypes that commonly can be confounded with asthma. The first, involves emotional stress, extrinsic or intrinsic irritants such as chemicals or spontaneous occurrence without a known trigger. It can easily be confused with asthma given its episodic nature and because stridor can sound similar to wheezing. Exercise-induced VCD also known as exercise-induced laryngeal obstruction (EILO), is not a manifestation of anxiety. It tends to occur in adolescent athletes. In these cases, a continuous laryngoscopy during an exercise (CLE) test is recommended for the assessment not only of VCD but also of laryngeal obstruction at the supraglottic level [25, 26].

### Obesity

Obesity is commonly associated with asthma. Obesity assessed by body mass index (BMI), is correlated with persistent low-grade systemic inflammation as adipose tissue is a source of cytokine production. It has been demonstrated that high-sensitivity C-reactive protein (hsCRP), tumor necrosis factor-alpha (TNF-a) and interleukin (IL)-6 concentrations are higher in obese than in non-obese individuals [27].

Obesity-associated asthma is a phenotype of asthma, characterized by decreased lung volumes, more pronounced respiratory symptoms, non-eosinophilic airway inflammation and generally poorer asthma control [5]. Respiratory symptoms associated with obesity can mimic asthma. In patients with dyspnea on exertion, it is important to confirm the diagnosis of asthma with objective measurement of variable airflow limitation [28]. A recent study showed that misdiagnosed asthma in obese subjects is due to an increased perception of dyspnea due to development of an excessive ventilatory response secondary to metabolic demands [29]. A more controversial explanation for increased dyspnea in obesity is its association with increased oxidative stress, which plays an important inflammatory role [30]. It has been demonstrated over time that weight reduction significantly improves the respiratory symptoms exhibited by obese patients [31–34].

### Obstructive sleep apnea

Obstructive sleep apnea (OSA) is a frequent condition associated with obesity and has been considered to possibly influence asthma [35, 36]. Furthermore, OSA has been associated with increased airway hyperresponsiveness [37]. It is associated with an upper airway inflammatory process that has the potential to influence the lower airways. Some investigators have reported bronchial neutrophilia and high IL-8, IL-6 and TNF-a concentrations in patients with untreated OSA. Continuous positive airway pressure treatment (CPAP) and weight loss improves symptoms and quality of life [38, 39].

### Dysfunctional breathing or “hyperventilation syndrome”

This condition may affect up to 10% of the population and is more prevalent in women and asthma patients [40]. Attacks of hyperventilation can be confused with asthma, both in patients with and without an actual asthma diagnosis. Patients frequently over-breathe or have an increased respiratory rate. Hyperventilation syndrome occurs when minute ventilation exceeds metabolic demands, resulting in respiratory alkalosis leading to dyspnea and chest tightness. It may often occur during acute anxiety, mimicking asthma [41, 42]. Spirometry at the time the patient is symptomatic can help distinguish the perception of dyspnea associated with a hyperventilation attack from asthma. There is no criterion standard to establish the diagnosis, however, in the presence of normal pulse oximetry during ambient air respiration a blood-gas measurement that demonstrates a low PCO2 and high pH at the time of symptoms provides more supportive evidence for hyperventilation. Breathing retraining by a physiotherapy intervention and anxiolytic therapy can significantly improve health-related quality of life scores [43].

### Gastroesophageal reflux disease

Gastroesophageal Reflux Disease (GERD) is a common cause of respiratory symptoms in adults. Generally, GERD is associated with post nasal-drip and often presents as a chronic cough (upper-airway cough syndrome) with chest tightness that is usually not associated with airway obstruction [44]. When it masquerades as asthma, additional symptoms such as heartburn and regurgitation may be present. Otherwise, GERD is often silent, and patients frequently deny symptoms. Differentiating cough variant asthma from GERD can be difficult because both frequently occur intermittently during the day and night [45]. In addition, its influence on asthma may be quite variable between patients but if it is suspected, it should be properly treated with a proton pump inhibitor or H2-antagonist and its effects on asthma carefully assessed. Currently, an empiric treatment course with a proton pump inhibitor for 4–8 weeks is recommended to determine if GERD is contributing to patients manifesting with cough or other unexplained lower respiratory symptoms [46, 47]. Other diagnostic approaches that are sometimes warranted include a 24-h pH probe which monitors the amount of acid in the esophagus over time. Lifestyle changes such as weight loss, not eating 2–3 h before bedtime and sleeping with the head of the bed elevated can also improve symptoms. In severe cases where the esophageal sphincter does not close, fundoplication surgery may be necessary [48].

## Psychological conditions: Depression, anxiety, symptoms subjective perception

Respiratory symptoms in patients without any known respiratory disorders can sometimes be a challenge for clinicians as it is usually a diagnosis by exclusion. Several studies have found a high prevalence of respiratory symptoms in psychiatric disorders, such as chest pain or discomfort, shortness of breath, or even the fear of dying [49, 50]. Dyspnea is a multidimensional construct with multiple sensations, such as suffocation sensation, hunger for air, uncoordinated breathing, or breathing that requires effort. Further investigation of these sensations may increase understanding of the cognitive control of breathing [51, 52]. Anxiety disorders are the most common mental disorders, followed by depression and panic disorders [53]. A nocturnal panic attack is a sudden awakening with a strong anxiety, usually after 2 h of sleep. During these attacks, there are prominent respiratory symptoms such as dyspnea, chest pain and choking [54]. Treatment consists of anti-anxiolytic medication like benzodiazepines, tricyclic antidepressants and psychotherapy [55].

## Intrathoracic mimickers of asthma

### Lower respiratory tract

#### Central Airway Obstruction

Central airway obstruction (CAO) refers to the obstruction of air flow in the trachea and the mainstem bronchus which may be malignant (primary broncho-pulmonary malignancies, endobronchial metastatic disease, mediastinal malignancies, lymphomas, laryngeal, esophageal carcinoma) or non-malignant (lymphadenopathy, vascular, surgical, traumatic, infectious). The luminal invasion can be extrinsic, intrinsic or mixed. Signs and symptoms develop when the CAO impairs airflow to the point of increasing the work of breathing or altering cardiopulmonary interactions. Some patients may be asymptomatic if the airway obstruction is mild, or they may be misdiagnosed as an asthma exacerbation due to symptoms of wheezing and dyspnea secondary to increased production of mucus, often associated with a respiratory tract infection, that improves with therapy. The presence of persistent unilateral wheezing should always prompt the investigation of focal airway obstruction. Stridor is a sign of severe laryngeal or tracheal obstruction. Patients may also present with other nonspecific symptoms such as exertional dyspnea and positional wheezing. With an anatomically fixed obstruction, shortness of breath and wheezing are typically unresponsive to bronchodilators; failure of a patient to improve with these measures should prompt the physician to consider the presence of CAO [56]. The evaluation of these subjects should always begin with spirometry, and it is particularly important to evaluate the shape of the flow-volume loop, in addition to the FEV1, FVC, and FEV1/FVC ratio, lung volumes and diffusion capacity. A flattened expiratory phase of the flow volume loop can be indicative of central airway obstruction [57]. A chest computed tomography (CT) scan is preferable over a chest x-ray as it provides more information about structural abnormalities. Bronchoscopy is usually necessary to assess the cause of airway obstruction [58]. For unstable patients, securing the airway with endotracheal intubation may be necessary. Several treatment modalities have been described depending on the cause and extent of involvement including bronchoplasty, electrosurgery, argon plasma coagulation, photodynamic and laser therapy, cryosurgery, airway stents and surgical resection [59].

#### Dynamic airway collapse

This process presents with cough, dyspnea and recurrent infections and is often misdiagnosed as asthma. It can be localized or diffuse and congenital or acquired. Tracheomalacia affects the trachea whereas bronchomalacia affects the bronchi and tracheobronchomalacia affects both. These are conditions where there is weakness of the tracheal and bronchial walls due to loss of integrity of cartilage and hypotonia of myoelastic elements. Tracheobronchomalacia is to be suspected when there is presence of monophonic wheeze that persists for longer than 10–14 days after every respiratory tract infection, wheezing that is worse when the child is awake and active and minimal when asleep, and when there is associated stridor [60]. In addition, this condition is minimally responsive to treatment with standard asthma medications. Dynamic airway collapse should be suspected in patients with a history of chronic bronchitis, prolonged intubation, surgery, post lung transplantation or cancer [61]. Diagnosis is confirmed if there is evidence of airway lumen narrowing by at least 50% with expiration on bronchoscopy or a CT scan [62–64]. First line treatment includes vocal cord exercises and pneumatic stenting with continuous positive airway pressure (CPAP) [65]. Stents can be used if there are flow limiting segments and surgery can be considered in severe cases [66].

#### Primary tracheal papilloma

This is a rare endotracheal benign tumor that can mimic asthma. Clinical symptoms of tracheal papilloma depend on the type of tumor, extent of endotracheal obstruction, and the degree of distal parenchymal damage. The most common physical finding is airway obstruction manifested by stridor, wheezing or rhonchi, chronic cough, dyspnea on exertion but usually the physical examination is normal. Repeated respiratory infection, intermittent hemoptysis, and blood-stained sputum can be present [67]. CT scan followed by endoscopy are the tests necessary for making the diagnosis. Endoscopic papillectomy is a safe and effective treatment and should be considered as first-line therapy for tracheal papilloma. Electrosurgery with snare loop is a short safe procedure performed using flexible bronchoscopy requiring only local anesthesia [68].

### Distal airway

#### Bronchiolitis obliterans

This is a nonspecific clinicopathologic syndrome involving the terminal airways resulting in dyspnea, dry cough, wheezing and progressive airflow obstruction [69]. The causes are multiple and differ between children and adults. A variety of diseases including post respiratory infections (e.g. RSV, cytomegalovirus, adenovirus, measles, varicella), toxic inhalation exposures, drug-induced, associated diseases (organ transplant, rheumatic diseases) and idiopathic bronchiolitis obliterans, may result in the development of bronchiolitis obliterans. Irreversible intrathoracic airflow obstruction and decreased defusing capacity of the lung for carbon monoxide (DLCO) can be observed. The diagnosis often requires a CT scan during expiration and a video-assisted thoracoscopic lung biopsy [70, 71].

#### Bronchiectasis

Bronchiectasis is characterized radiologically by permanent dilatation of medium-size bronchi, and clinically manifests as a persistent cough with sputum production and recurrent lower respiratory infections [72]. Disorders associated with bronchiectasis such as cystic fibrosis (CF), common variable immunodeficiency (CVID) and allergic bronchopulmonary aspergillosis (ABPA) should be excluded by ordering appropriate testing. For example, for CVID it is recommended to obtain quantitative immunoglobulin (IgA, IgM, IgG) and if IgG is low, pre- and post-pneumococcal 23 vaccination to determine if the patient’s B-lymphocytes are producing adequate IgG responses should be performed. Patients with ABPA typical exhibit central bronchiectasis. Excluding ABPA as a cause of bronchiectasis requires obtaining a total IgE level, immediate cutaneous reactivity to *Aspergillus fumigatus*, peripheral blood eosinophil count, and serum specific IgE antibodies to the recombinant *A. fumigatus* allergens rAsp f 1, 3, 4 and 6 [73, 74]. Sputum culture to identify *A. fumigatus* and to exclude non-tuberculous mycobacterial (NTM) infection should be obtained. Measurement of serum IgA antibody to glycopeptidolipid core antigen specific for mycobacterium avium complex (MAC) may also be indicated. Testing for CF with a sweat chloride screening test is recommended for patients aged < 40 years with recurrent *Pseudomonas aeruginosa* and *Staphylococcus aureus* infection. High resolution chest CT can confirm the presence of central bronchiectasis. The goals of bronchiectasis therapy are to improve airway mucus clearance through physiotherapy, use of mucolytics and other devices like a flutter device and compression vest. The objective is to suppress, eradicate and prevent airway bacterial colonization and reduce airway inflammation in order to improve QoL [75].

#### Chronic obstructive pulmonary disease (COPD)

COPD can be difficult to differentiate from asthma because both present with dyspnea, wheezing, chest tightness, cough and airflow obstruction. However, COPD is more typically categorized as fixed obstruction in adult smokers. In contrast, asthma patients often demonstrate complete reversibility of airflow obstruction. However, patients with COPD can have airway hyperresponsiveness and reversibility, and patients with chronic asthma can develop fixed obstruction. In addition, both diseases are common, especially in the elderly, so they may coexist which is referred to as asthma COPD overlap (ACO) [4]. Some may argue that distinction between COPD and asthma is not necessary, as the underlying physiology and goals of treatment are similar: a combination of ICS and long-acting β_2_-adrenergic agonist can improve patient outcomes [76] However, although they are both characterized by chronic inflammation, the initial triggers and type of inflammatory response differ as does clinical management and prognosis [5]. The most common phenotypes of COPD are emphysema and chronic bronchitis which present more commonly in older patients and alpha 1-antitrypsin deficiency which typically presents at a younger age. Both asthma and COPD patients report symptoms with exercise, but COPD typically is related to increased oxygen demand and often associated with hypoxemia. Cardiac failure due to pulmonary hypertension may coexist in patients with end-stage of COPD. A lower DLCO and increased prevalence of chest CT scan abnormalities may help distinguish COPD from asthma. Pulmonary function testing should be performed after any acute exacerbations have been adequately treated and the patient has been stabilized to determine the degree of reversibility that can help differentiate COPD from asthma [77].

#### Tuberculosis

Endobronchial tuberculous (TB) and TB lymph nodes causing extrinsic airway compression can initially present with wheezing but are usually accompanied by fever. An isolated cough is rarely a manifestation of TB [78].

### Other intrathoracic considerations

#### Cardiac heart failure

Left ventricular heart failure or pulmonary venous obstruction can lead to pulmonary vascular distention, airway edema and wheezing. An enlarged pulmonary artery or dilated left atrium can cause extrinsic compression of the airways, leading to monophonic wheezing. In children, cardiac heart failure (CHF) rarely mimics asthma. Physical examination will reveal the underlying cause based on clinical features of heart failure such as cyanosis, heart murmurs, gallop rhythm. Paroxysmal nocturnal awakening with dyspnea and orthopnea may improve with standing or sitting. On the other hand, exercised induced symptoms are associated with increased oxygen demand similar to COPD. A chest X-ray, 12-lead ECG and echocardiography should be performed if CHF is suspected [79].

#### Accidental foreign body

This can occur in all ages but is more frequent among children under 2 years of age. The most frequent manifestations are cough, wheeze and respiratory distress and sometimes airway collapse. Chest radiographs may show evidence of air trapping or atelectasis of distal lung parenchyma [80] bronchoscopy helps both in diagnosis and in retrieving the foreign body [81].

#### Cystic fibrosis (CF)

Cystic fibrosis is the most common fatal hereditary airway disease among Caucasians and can show symptoms at a very young age [82]. The classical presentation of gastrointestinal malabsorption may not be present, and some patients will not present with clinical symptoms until adolescence or early adulthood. Cystic fibrosis should be suspected when signs of airway disease persist despite high-dose systemic corticosteroids. The most important clinical features are persistent wet productive cough, recurrent chest infections, finger clubbing and failure to thrive. Patients with CF often experience associated recurrent sinusitis. The gold standard for diagnosis remains the sweat chloride test but DNA based tests may be necessary especially when the sweat chloride test is equivocal. Early diagnosis of CF is important to improve long term clinical outcomes. Management of the pulmonary issues includes early institution of airway clearance techniques, aggressive treatment of infections with antibiotics and close follow-up monitoring [83].

#### Primary ciliary dyskinesia

Primary ciliary dyskinesia is an autosomal recessive disease, characterized by motile ciliary dysfunction with symptoms starting soon after birth. A delayed diagnosis of this condition is common. These patients develop chronic wet cough, which can be exacerbated by viral infection, mimicking chronic asthma. Genetic analysis and next generation sequencing is quickly becoming the recommended early diagnostic tool. High speed video microscopy analysis and transmission electron microscopy of nasal epithelial cells to evaluate ciliary ultrastructure has also been used to diagnose this condition [84, 85]. The definitive diagnosis is still in a state of flux. A combination of tests have been proposed such as nasal nitric oxide (nNO) and FeNO measurements which have good sensitivity and low specificity in PCD diagnosis. Patients with PCD are known to have an abnormal nNO value which may serve as a useful screening test for this diagnosis [86, 87].

#### Metastatic carcinoid

Metastatic carcinoid syndrome is caused by secretion of neuropeptides from neuroendocrine tumors and presents as episodic flushing, hypotension and wheezing. Carcinoid tumors that arise in the airway lead to severe airway obstruction that results also in wheezing and chronic dyspnea, even obstructive pneumonia. Diagnosis of carcinoid can be challenging but patients often have increased 24-h urine levels of 5-Hydroxyindoleacetic acid (5-HIAA), increased chromogranin a (a secretory protein found in neuroendocrine cells) levels in serum and a positive somatostatin (octreotide) receptor scintigraphy test. A chest CT scan should be performed to identify the tumor. Fibrotic bronchoscopy and biopsy is typically necessary to confirm the diagnosis histologically [88].

#### Lymphangioleiomyomatosis

This is a chronic, progressive and obstructive airway disease that affects women of childbearing age. It presents with progressive dyspnea and cough, wheezing, chest pain complicated by chylous pleural effusions, hemoptysis and pneumothoraxes. The diagnosis is often initially missed, as it can confound with asthma. Transbronchial biopsy specimens in conjunction with staining for HMB 45, a monoclonal antibody with specific immunoreactivity for malignant melanoma but also for smooth muscle cells, are recommended for confirming a diagnosis of LAM. The management of the disease consists of supportive treatment, hormone therapy and in severe cases, lung transplantation [89].

#### Hypersensitivity pneumonitis (HP)

Hypersensitivity pneumonitis also known as extrinsic allergic alveolitis, is a complex clinical syndrome of nonspecific pulmonary symptomatology resulting from recurrent antigen exposure, IgG activation, and alveolar lymphocytosis. Known toxic antigens include inhaled mycobacterial, bacterial, fungal, animal protein, and chemical substances although the search for an inducing cause may not be identified in up to 60% of patients. Hypersensitivity pneumonitis occurs in both, atopic and non-atopic individuals. The diagnosis consists of recognizing the clinical symptoms (acute fever, weight loss, rales) in correlation with exposure history (usually occupational). Diagnosis of HP is confirmed with laboratory testing (serum precipitins, quantitative immunoglobulins, lactate dehydrogenase-LDH, erythrocyte sedimentation rate-ESR, C-reactive protein, antinuclear antibodies-ANA) and the presence of characteristic radiographic abnormalities most notably patchy, peripheral, bilateral interstitial infiltrates in the acute phase and fibrosis in the chronic form [90]. Treatment beyond antigenic avoidance is primarily limited to corticosteroid therapy and sometimes immunosuppressant agents for the chronic form [91].

#### Sarcoidosis

Sarcoidosis is a multisystem inflammatory disorder, characterized by accumulation of large numbers of interleukin 2 (IL 2) releasing helper (Leu-3+) T cells in involved organs, noncaseating granulomatous inflammation in the lung, lymph nodes, skin and other affected tissues. More than 90% of patients present with lung manifestations such as cough, dyspnea on exertion or chest pain, airways hyperresponsiveness and obstruction [92]. The diagnosis of sarcoidosis depends on association of clinical symptoms compatible with radiographic findings, pulmonary function tests that include a DLCO, laboratory test results such as hepatic and renal panels, measurement of serum calcium, and an ophthalmologic examination. Elevation of angiotensin-converting enzyme (ACE) levels in sarcoidosis appears to be associated with the active disease process [93]. The first line of treatment for sarcoidosis is corticosteroids, but in cases of intolerance or side effects, second line treatments have included methotrexate, azathioprine, leflunomide, and infliximab [94].

### Intrinsic and extrinsic eosinophilic disorders

Uncommon asthma masqueraders include tropical eosinophilia, Loeffler’s syndrome, hypereosinophilic syndrome and allergic angiitis. A detailed history and examination and appropriate laboratory investigations will usually reveal the appropriate underlying diagnosis.

#### Tropical pulmonary eosinophilia

This is a rare syndrome, characterized by a persistent or recurrent cough worse at night and is associated with generalized weakness, shortness of breath, weight loss, low grade fever, and splenomegaly. Wheezing may be an associated symptom. It is caused by *Wuchereria bancrofti*, a filarial infection, most frequently found in Asia. The diagnosis is established by a history of exposure, the presence of peripheral eosinophilia and increased titers of anti-filarial antibodies. Chest radiograph abnormalities include diffuse miliary lesions, cavitation or patchy consolidations and reticulonodular infiltrates. A good response to diethylcarbamazine, an inhibitor of arachidonic acid metabolism in microfilaria, is very supportive of the diagnosis [95].

#### Eosinophilic Granulomatosis with Polyangiitis

This is a necrotizing, antineutrophil cytoplasmic antibody (ANCA) vasculitis associated with eosinophilia with a predilection for involving the respiratory mucosa [96]. There are three distinct clinical phases. The prodromal phase consists of rhinosinusitis and asthma, which may exist for several years until diagnosis This is followed by the development of peripheral eosinophilia with myocardial, gastrointestinal, and pulmonary involvement. The final phase is a progression to systemic vasculitis with development of dermatologic, neurologic, and renal involvement. This syndrome remains a clinical diagnosis which is confirmed by radiologic, laboratory, and pathologic evidence. Four of the following six criteria must be satisfied to establish a diagnosis. These criteria include asthma, peripheral eosinophilia > 10%, mono or polyneuropathy, paranasal sinus abnormality, nonfixed pulmonary infiltrates and tissue biopsy containing a blood vessel with extravascular eosinophils. Therapy consists of oral corticosteroids but for more severe progressive cases, cyclophosphamide, cyclosporine, interferon alpha, mycophenolate mofetil and azathioprine have been used with variable success [97].

#### Hypereosinophilic syndrome

Hypereosinophilic syndrome (HES) is a group of rare blood disorders with a high number of eosinophils. The most common respiratory symptoms are cough, shortness of breath, fatigue and fever. The diagnosis can be challenging because the symptoms are commonly seen with other allergic diseases and medical conditions. Thus, the first step is to rule out other underlying conditions [98]. The standard treatment remains corticosteroids and chemotherapeutic agents such as hydroxyurea, chlorambucil and vincristine. The new approved drugs such as tyrosine kinase inhibitors (imatinib) and monoclonal antibodies including anti-IL5 or IL5R, anti-IL4alpha and anti CD25 seem to be the future treatments of HES [99, 100].

#### Loeffler Syndrome

Loeffler syndrome is characterized by transient pulmonary infiltrates associated with dry cough, dyspnea, low grade fever, rales, wheezing and fleeting migratory eosinophilia in patients infected with helminth larvae such as *Ascaris* and *Strongyloides* This condition typically resolves spontaneously within 4 weeks [101].

## Conclusion

The symptoms of asthma may represent a variety of underlying pathologic processes supporting the adage that *not all that wheezes is asthma and all that does not wheeze is not asthma*. Clinicians must maintain a high index of suspicion for diseases that mimic asthma, especially when patients present atypically or fail to respond to therapy. In these cases an extended differential diagnosis must be considered. Through these efforts, symptomatic relief may be achieved in a greater proportion of patients suspected to have asthma, thereby improving their quality of life and reducing unnecessary treatment expenditures.

## Key points

1. Asthma can be a difficult condition to correctly diagnose.
2. Clinicians should entertain alternative conditions especially when patients are unresponsive to conventional asthma medications.
3. Asthma mimickers can be extrathoracic or intrathoracic.
4. Other more common mimickers of asthma include pulmonary eosinophilic disorders, sarcoidosis, hypersensitivity pneumonitis, CF and CHF.
